# Physical Culture for Children

**Published:** 1902-11

**Authors:** Ida Richards Compton


### PHYSICAL CULTURE FOR CHILDREN.*

By IDA RICHARDS COMPTON.

A noted philosopher said: "It is possible to be seventy years young instead of forty years old."

This leads us to ask, what is youth? What is old age?

*Read before the Georgia Sociological Society, Atlanta, June, 1902.

Youth is emblematic of newness, freshness, life, growth, vigor, strength, health and beauty. And ever since the "fountain of youth" was lost in the Garden of Eden mankind has continued in search of it. But as Ponce de Leon sought in vain for waters which would give him perpetual youth, so all men have failed to find any magic key which would lock out old age and its aftermath.

And why? Because perpetual youth is impossible, or because it is sought in the wrong way?

The answer comes, both. First, because old age and death are the result of sin in the beginning, and must come to every one. Second, because the average man brings upon himself and his posterity premature signs of old age and approaching death by disobeying the most simple laws of health.

The average man evidently thinks that the "fountain of youth" is to be discovered in a drug-store. If the quack doctor could administer his "Resurrection Pills," or "Elixir of Life" to the great enemy Death, with the promised results, then indeed one might hope to live forever and always remain young and vigorous and in perfect health.

"It is the vast field of ignorance pertaining to these subjects in which quackery thrives and fattens."

Why is it that almost a fourth part of the children that are born die before they are one year old? Why is it that more than one third die before they are five years of age? And still more incredible, why do more than one half of all that are born die before they are eight years of age? Is it the fault of the children, or are they responsible for this? No, indeed, it is the fault of the parents or guardians. A child's natural appetites and instincts are warped and distorted by its environments as well as inherited tendencies.

When a child is born it immediately breathes and moves and soon cries for the nourishment nature intends for it. And as it grows, the one thing a child does during almost every waking moment is to keep in motion. And, when old enough to follow its own inclinations, it seeks the sunshine, fresh air and the invigorating touch of the electrified earth. How happy a child is when seated upon a pile of sand, or making mud pies, while his lusty lungs are drinking in pure oxygen and his body is invigorated by the penetrating sun's rays. How delighted he is when paddling in a puddle of water, rolling upon the grass or playing among flowers, especially in the wild woods, over hill and dale and by the babbling brook. And how the growing young body enjoys running, jumping, climbing or throwing any object the child is able to handle.

And children's natural appetites crave only that food which they are able to digest, until distorted by being tempted with poisonous foods which their parents eat. I remember the case of an infant where the first thing ever given it to taste was coffee. She grew up to be a most delicate young lady, and is still a slave to barbarous customs.

Children teach us lessons regarding sleep, for, when the day is done, their weary little eyelids close in sleep until the morning light awakens them to the coming day, and nature has been given ample time to restore what has been torn down during the preceding day.

Nothing is more interesting than to watch a child in its physical development from the cradle up to the time it is placed in school. During a child's waking hours it is perfectly still for hardly a moment, and how it teaches us the lesson of exercise! And, during this age of freedom how rapidly children grow and develop if left to nature's care.

Then comes school life, in which so many of the child's natural tendencies, mental, moral and physical, are dwarfed and fettered. Especially is this the case with girls. Boys are allowed more freedom for physical development in their sports connected with school life.

But the dear little girls, still under the curse of superstition and prejudice and custom, the moment they enter school must begin to learn to be intellectual little ladies; their little faces must not be allowed to be kissed by the sun for fear every kiss may leave a freckle; they must be so careful of every movement, lest their tresses lose their curl; they must be so careful not to get their spotless aprons soiled; they must not handle boyish things, lest their hands become enlarged; their feet must be bound up in tight shoes, lest they become too large; they must not run or jump or participate in boyish games, lest they become too boisterous and lose that refinement and delicacy of manner necessary in polite society; and before they have reached their teens the perfect young forms must be imprisoned in corsets so they may develop the figures decreed by fashion!

What nonsense! Such ideas are worse than nonsense! They are barbarous! They are even more, they are criminal!

And, as the average parent thinks and is, so the average child will think and be. For illustration, a little girl friend of mine, delicate in health, was walking by my side down the street. Her carriage was extremely stiff and ungraceful. I said to her, "You look as if you had on a tight, stiff corset."

"Oh, yes," she replied, "you know I am thirteen years old now, and it is time I was getting a good figure, so I have on my first corset."

I endeavored to explain to her some of the evils which would result therefrom, but, "no," she replied, "mother and sister never go without their corsets, so I must wear them, too.

Thus the sins of the parents are handed down to the children generation after generation, and, though such great responsibilities devolve upon parents in the rearing of their children, how little common sense is used regarding the laws of health.

The majority of mothers are so interested in their children's clothes, in the outward appearance, in the report cards regarding the standing in school, that they, to a great extent, lose sight of the true meaning of education.

From our superficial knowledge of the depth, breadth or height of anything in the mental, moral or physical man, or of the things of the wide world about which man desires to know, parents and teachers all fall far short of the best way to teach, to develop, to educate. For, every individual is differently constituted, mentally, morally and physically, from every other individual; therefore different methods, different leadings, different inspirations, are necessary for the highest development of each individual.

Every one knows that the foundation of many serious diseases is laid in the schoolroom. The causes are many, sometimes from a neglect of exercises; from too long confinement in one position or upon one study; from overexcitement and overstudy; from breathing bad air; from being kept too warm or too cold; from bad light upon the eyes; from too great a strain upon the nervous system and appealing to exciting motives. So often the young mind is taxed with subjects too many and too great for its comprehension at the time, and is not made to take the fresh air, exercise and recreation so necessary to its health, in order to press the pupil on to the accomplishment of a certain task by a certain time. We are so eager to develop a precocious intellect, oftentimes we "crush the casket in order to gratify a prurient desire to astonish the world with the brilliancy of the gem."

Well do I remember when in my last year at college, so great was my desire to graduate by a certain time and so meager was my knowledge of physiological laws and the results of disobeying them, and having no one to make me obey them, at the time assigned my class for an hour's daily walk, I took the demerits given for my absence, and spent the time in study. Each morning I arose early and studied some hours before breakfast. During the last six months spent in college I was never free from headache for one waking hour, and the day I graduated I was just on the verge of nervous prostration; and nothing but my immediate removal to the country, where, during the summer months, I became almost an athlete in my enjoyment of outdoor sports, saved me from a severe illness.

Had I been truly educated? No, for education is preparation for the complete living of the mental, moral and physical man.

If a child does not understand the reasons for needful exercise and should be inclined to be over studious, every inducement should be made to have it become interested in some form of systematic exercise. These should be outdoor sports where possible, and make them so attractive that the child will love them.

Or, if in cities where there are not the needed outdoor playgrounds, children should be given systematic exercise, especially enjoyable games, under a trained instructor in a fully equipped gymnasium. And, though all the exercise should be given according to physiological laws, and for the best possible improvement of the body, mind and soul, yet that is no cause for the exercises not being made as enjoyable as possible to the children. That which is healthfully enjoyable, inspiring and exhilarating brings the quickest, surest and most lasting results. All gymnasium work should be given by such a skilled instructor and one so adapted to the work, one so capable of interesting, inspiring and enthusing children, that they will regard the gymnasium as a delightful playground.

One of the greatest obligations resting upon parents is to have their children examined by a physician from birth on through the growing years at least twice a year, to see if they are being normally developed. From disease, a fall, incorrect standing, walking or sitting, weak muscles by inheritance, the habit of carrying books or some weight always on the same hip, or resting by always relaxing and lowering the same hip and shoulder, has developed in childhood curvature of the spine and its attendant evils in thousands of cases, which, if they had been examined and the tendency to the evils discovered in time, could have been permanently cured by exercise given under a competent instructor according to physiological laws, provided the parents aided in carrying out the right laws of living in the home and every-day life.

And, if we but notice the drooping shoulders and sunken chests of thousands of children everywhere, we can easily see how indigestion, nervousness, poor circulation and especially consumption are courted and the way made easy for deadly diseases to fasten themselves upon the body. Nothing but exercise, which of course includes correct breathing, will raise drooping shoulders and sunken chests.

Again, it is the sacred duty of every guardian, whether parent or teacher, to interest children under their care in the study of their own bodies, made in the image of God, and they should inculcate into their very being the importance of guarding their bodies as sacred temples for the indwelling of God's spirit.

If the parents and teachers of the present generation had labored as diligently to teach every child to know itself, as they did to teach it "reading, writing and arithmetic," we would have a higher order of human beings peopling our globe to-day.

It is the fault and ignorance of the teacher where the study of the "human body" proves dull and uninteresting, for it can be made the most fascinating and interesting of all studies, even to children. If appealed to in the right manner, many of our children would develop into artists, sculptors and scientists through the study of the wonderful mechanism, perfection and beauty of the human body as God would have it to be; and the world, at no distant day would become one great "Sociological Society," with God as the great President, and with each individual member at work with hand and heart, striving to hasten the millennial dawn, when we shall awake in His likeness, body, mind and soul.

No one but a physician or one who has made a careful study of the subject, has any conception of the effect of the different foods, not only directly upon the health of children, but upon their minds and hearts through the nervous system. We all know something of the effects of stimulants or intoxicants upon adults. So, but a moment's reflection will lead us to see how certain drinks, as coffee or tea, or foods that heat the blood, can easily form the foundation in children for appetites for stronger stimulants as they grow older. If much meat, especially pork, or rich foods, are given often to children (or adults, either), it will unduly heat the blood, irritate the nervous system, and cause them to be nervous, irritable and quarrelsome. This has been tested and proven. Children of ungovernable tempers who had been allowed to eat rich meats and indigestible foods three times a day, when such food was denied them and they were given a diet suited to their years, became as gentle and docile as lambs.

The need of physical culture for children should also be viewed from the standpoint of teaching them self-control. The rapidly growing boy, especially when he reaches the self-conscious age, finds himself greatly in his own way; and not knowing what to do with his hands or feet, he trips over the rugs, stumbles over the chairs, knocks over the dishes on the table, and blushes and stammers over his speech. Such a boy needs to have his body brought under subjection, and nothing will give him such self-control, such mastery over self, such ease of manner and grace of movement as a thorough course in physical training.

Then, too, many of our girls come to a self-conscious age. Whether they sit or stand or walk or talk, they seem so ill at ease they make every one about them uncomfortable, too. They imagine their clothes are on awry and must be pulled into place; a curl must be patted or a lock of hair smoothed into place; the position of the hat needs a little change; they feel their eyes batting too often, and therefore set them upon some certain spot until the tears almost come; the lips need moistening very often and repeatedly put in primp, as if to say "pie" or "pudding"; while such an added task as centering the mind upon any connected theme of conversation is simply impossible.

The carriage of the body either makes or mars perfection of form, greatly affects the whole nervous system, the circulation, respiration and digestion and, in fact, has great influence upon character. If you doubt this, just try an experiment on yourself. Hang your head, let the chest sink and the shoulders droop, extend the abdomen forward and press the ribs against it, let your arms fall limply forward, loosen the joints of your knees and rest the weight of your body on one leg, and I assure you if you will retain this graceful (?) and commanding (?) and elevating (?) posture for about two and one half minutes, you can easily feel yourself a "nobody," an "outcast" or one bereft of your last friend.

Then, raise your head and chest well up, let your arms fall naturally, extend the hips back, poise the weight of the body upon the balls of the feet instead of the heels, feel sure of your balance, brighten your face, take a deep inhalation of pure air (without lung restriction), and you will feel like exclaiming, "As a field for aiming toward all that is highest and best in uplifting myself and my fellow-man, the world is mine!"

Our bodies are but machines controlled by our minds. Therefore, cultivate cheerfulness if you would have a bright face; cultivate temperance in all things and peace will reign over you; cultivate hope, and by the light of it you will uplift others; cultivate love, and you may conquer the world about you, for all these within shine without and glorify the whole.

Know thyself as God would have you, and thou hast the art of living.

